# From “wanting to exercise” to “sticking with exercise”: motivational profiles and configurational pathways to high exercise adherence among college students—an LPA and fsQCA analysis

**DOI:** 10.3389/fpsyg.2026.1886854

**Published:** 2026-07-27

**Authors:** Jia Zheng, Xinyue Chang, Yawei Ren

**Affiliations:** 1 Sichuan Sports College, Chengdu, China; 2 Physical Education, Sichuan Normal University, Chengdu, China

**Keywords:** college students, exercise adherence, exercise motivation, fuzzy-set qualitative comparative analysis, latent profile analysis, self-determination theory

## Abstract

**Background:**

Exercise adherence is a key challenge in promoting sustainable physical activity among college students. Although self-determination theory has been widely used to explain exercise motivation, less is known about how different forms of motivation coexist within individuals and combine to support high exercise adherence.

**Objective:**

This study aimed to identify latent exercise motivation profiles among Chinese college students and examine motivational configurations associated with high exercise adherence.

**Methods:**

A cross-sectional survey was conducted among 2,677 college students from 20 universities in Sichuan Province, China. Exercise motivation was measured using the Chinese-translated Behavioral Regulation in Exercise Questionnaire-2, and self-reported exercise adherence was assessed using the total score of the Exercise Adherence Questionnaire. Latent profile analysis was used to identify motivational profiles based on amotivation, external regulation, introjected regulation, identified regulation, and intrinsic motivation. The Bolck, Croon, and Hagenaars method was used to compare exercise adherence across profiles, with Holm-Bonferroni adjustment for pairwise comparisons. Fuzzy-set qualitative comparative analysis was used to identify motivational configurations associated with high and non-high exercise adherence.

**Results:**

Four latent motivation profiles were identified: intrinsic-dominant motivation profile, amotivated–controlled motivation profile, high autonomous motivation profile, and identified-regulation dominant profile. Exercise adherence differed significantly across the four profiles. After Holm-Bonferroni adjustment, students in the high autonomous motivation profile and intrinsic-dominant motivation profile showed higher exercise adherence than those in the amotivated–controlled motivation profile and identified-regulation dominant profile, whereas the two higher-adherence profiles did not differ significantly from each other. The fsQCA results indicated that no single motivational condition was necessary for high exercise adherence. One sufficient configuration for high exercise adherence was identified: the absence of amotivation, the absence of external regulation, and the presence of intrinsic motivation. Non-high exercise adherence was explained by the opposite configuration.

**Conclusion:**

Exercise adherence among college students is associated with both motivational profiles and motivational configurations. Promoting adherence requires reducing amotivation and external control while strengthening intrinsic motivation, enjoyment, autonomy, and personally meaningful exercise experiences.

## 1 Introduction

### 1.1 Exercise adherence among Chinese college students

Promoting regular physical activity has become an increasingly important public health and educational priority in China. Under the broader policy framework of Healthy China 2030, physical activity is no longer treated merely as an individual lifestyle choice, but as a key component of national health development, disease prevention, and quality-of-life improvement (State Council of the People’s Republic of China, 2016). The Healthy China Action (2019–2030) explicitly emphasizes the importance of improving physical fitness, promoting active lifestyles, and creating supportive environments for health behavior change (General Office of the State Council, 2019). In parallel, the Composing and Editorial Board of Physical Activity Guidelines for Chinese (2022) provides population-specific recommendations and identifies regular movement, reduced sedentary behavior, and long-term persistence as core principles of physical activity promotion in the Chinese context. Together, these policies highlight a central concern for universities: how to help young adults develop sustainable exercise habits before they enter full adulthood (Wang et al., 2022).

Chinese college students represent a particularly important population for exercise promotion. University years are a transitional period during which students gain greater autonomy over their time, daily routines, social relationships, and health behaviors. In principle, this autonomy should create opportunities for regular physical exercise. In practice, however, many Chinese college students face academic pressure, examination demands, employment uncertainty, irregular schedules, digital entertainment, and uneven access to sport facilities or professional exercise guidance (Brown et al., 2024; Pan et al., 2022). A systematic review of physical exercise among Chinese college students found that lack of time caused by academic pressure, limited facilities, and insufficient professional guidance are important barriers to exercise participation (Pan et al., 2022). These findings suggest that the problem is not simply whether college students know that exercise is beneficial; rather, the more difficult issue is whether they can maintain exercise over time in the face of competing demands (Han et al., 2025; Zhang D. Y. et al., 2025).

For this reason, exercise adherence is a particularly meaningful outcome in the university context. Compared with occasional exercise participation or general physical activity level, exercise adherence captures a more stable and behaviorally important question: whether students can persist in exercise despite difficulties, distractions, emotional fluctuations, and environmental barriers. In other words, the core challenge is not merely moving students from inactivity to activity, but helping them move from “wanting to exercise” to “sticking with exercise.” Recent research among Chinese college students has also treated exercise adherence as a psychologically meaningful health behavior. For example, Liu et al. (2024) examined exercise adherence in relation to peace of mind among Chinese college students, highlighting the relevance of adherence not only for physical health, but also for psychological wellbeing.

### 1.2 Exercise motivation from the perspective of self-determination theory

Self-determination theory provides a useful framework for explaining why some students are more likely to persist in exercise than others (Ryan and Deci, 2017). A central proposition of self-determination theory is that motivation differs not only in quantity, but also in quality. According to self-determination theory, exercise participation may be driven by qualitatively different forms of regulation, ranging from amotivation and external or introjected regulation to identified regulation and intrinsic motivation. Ryan and Deci argued that human motivation varies along a continuum of self-determination, from controlled or non-self-determined motivation to more autonomous forms of regulation. More self-determined forms of motivation are generally expected to support more adaptive, persistent, and self-endorsed behavior (Murphy and Taylor, 2022; Teixeira et al., 2012; Vansteenkiste et al., 2007; Yan et al., 2025).

In the exercise domain, the Behavioral Regulation in Exercise Questionnaire-2 was developed to assess different forms of exercise motivation based on self-determination theory (Markland and Tobin, 2004). Markland and Tobin modified the original BREQ by reinstating amotivation items, thereby allowing the instrument to assess a broader motivational continuum in exercise contexts. The BREQ-2 includes five motivational regulations: amotivation, external regulation, introjected regulation, identified regulation, and intrinsic motivation. These dimensions are especially relevant for studying college students because university exercise behavior may be shaped by both autonomous and controlled reasons, as well as by the absence of meaningful reasons for participation (Chen et al., 2008).

Importantly, the Chinese-translated BREQ-2 has been validated in Chinese university contexts. Liu et al. (2015) examined the psychometric properties of the Chinese-translated Behavioral Regulation in Exercise Questionnaire-2 among university students from Mainland China and Hong Kong. Their findings supported an 18-item, five-factor structure and provided evidence for factorial validity, discriminant validity, nomological validity, internal reliability, and measurement invariance across the two Chinese university samples. This makes the C-BREQ-2 particularly appropriate for examining exercise motivation among Chinese college students.

### 1.3 From single motivational effects to motivational profiles

Although self-determination theory has been widely used to study exercise motivation, much existing research still follows a variable-centered logic. Such studies typically examine whether intrinsic motivation, identified regulation, introjected regulation, external regulation, or amotivation has a positive or negative association with exercise behavior (Gao et al., 2025). This approach is useful for estimating the average effect of each motivational variable, but it may oversimplify the motivational reality of college students. In everyday life, motivational regulations often operate together rather than in isolation: enjoyment may coexist with body-image concerns, perceived health value, peer expectations, or self-imposed obligation. Thus, different forms of motivation can coexist within the same individual (Ostendorf et al., 2021; Vlachopoulos and Karageorghis, 2005).

This coexistence of multiple motivations suggests the need for a person-centered perspective. Instead of asking only whether a single type of motivation predicts exercise adherence, it is necessary to ask what motivational profiles exist among Chinese college students. For example, some students may show high intrinsic motivation and high identified regulation, forming an autonomous motivation profile. Others may show high identified regulation together with high introjected regulation, forming a mixed motivation profile. Some may be driven primarily by external regulation, whereas others may show high amotivation and low self-determined motivation (Gao et al., 2025). These different motivational patterns may have different implications for exercise adherence, even when their average motivation scores appear similar (Liu and Wei, 2026).

Latent profile analysis is well suited for this purpose. LPA is a person-centered method that identifies unobserved subgroups based on individuals’ patterns across multiple continuous indicators. Rather than assuming that all students follow the same average motivational structure, LPA allows researchers to uncover heterogeneous motivational profiles within the student population. Methodological reviews emphasize that LPA is particularly useful for identifying meaningful subgroups that may be hidden in traditional variable-centered analyses and for studying complex patterns of individual differences. In the present study, LPA is used to identify latent exercise motivation profiles based on the five C-BREQ-2 dimensions: amotivation, external regulation, introjected regulation, identified regulation, and intrinsic motivation.

Previous exercise motivation research has begun to demonstrate the value of this person-centered logic. Lindwall et al. (2017), for example, identified within-person latent profiles of exercise motivation and showed that different behavioral regulations can combine into heterogeneous motivational patterns. Harbichová et al. (2025) similarly identified distinct sport motivation profiles among Czech university students, further demonstrating motivational heterogeneity in university populations. Related work has also emphasized that less self-determined motivational processes are relevant for understanding intention to continue exercise and potential dropout risk (Rodrigues et al., 2019). Together, these studies suggest that exercise motivation is better understood as a multidimensional pattern than as a set of isolated predictors. However, less is known about how such motivational profiles are associated with exercise adherence among Chinese college students, and profile analysis alone does not specify which combinations of motivational conditions are sufficient for high exercise adherence.

### 1.4 Configurational pathways to high exercise adherence

However, identifying motivational profiles alone is not sufficient. LPA can answer the question of “what types of students exist,” but it does not fully explain how particular motivational conditions work together to support high exercise adherence. In the university context, high adherence may depend not only on whether students enjoy exercise or recognize its value, but also on whether amotivation and external regulation are relatively weak. In this sense, exercise adherence may be shaped by conjunctural causation, where the meaning and effect of one motivational condition depend on the presence or absence of other conditions.

This issue is especially important in the Chinese university context. Chinese college students often experience exercise as both a personal health behavior and a socially embedded activity. Exercise may be shaped by individual interest, health awareness, body-image concerns, academic schedules, peer relationships, campus sport culture, and institutional expectations (Cui et al., 2026; Shi et al., 2026; Zhang L. et al., 2025; Zuo et al., 2025). As a result, the association between any single motivational regulation and adherence may depend on the broader motivational configuration in which that regulation is embedded. For instance, introjected regulation may be associated with psychological pressure when it appears alongside high amotivation, but it may function differently when combined with strong identified regulation and low amotivation. Similarly, external regulation may not necessarily have the same implication across all students if it is accompanied by personal value recognition or a supportive exercise environment. Such possibilities are difficult to capture through conventional regression-based models that focus on the net effect of isolated predictors (Jia et al., 2025).

Fuzzy-set qualitative comparative analysis provides a useful configurational approach for examining this complexity. fsQCA is grounded in set theory and is designed to identify combinations of conditions that are sufficient for an outcome. Unlike conventional variable-centered methods, fsQCA emphasizes conjunctural causation, equifinality, and causal asymmetry. Ragin’s work established fuzzy-set analysis as a way to understand cases as configurations of conditions rather than as independent variables operating in isolation (Ragin, 2008, 2009). Fiss further showed that fsQCA can be used to build stronger causal theories by distinguishing core and peripheral conditions within configurations (Fiss, 2011). For the present study, fsQCA allows the five motivational dimensions of the C-BREQ-2 to be treated as distinct condition sets and makes it possible to examine whether one or more motivational configurations are sufficient for high exercise adherence.

### 1.5 The present study

**FIGURE 1:**
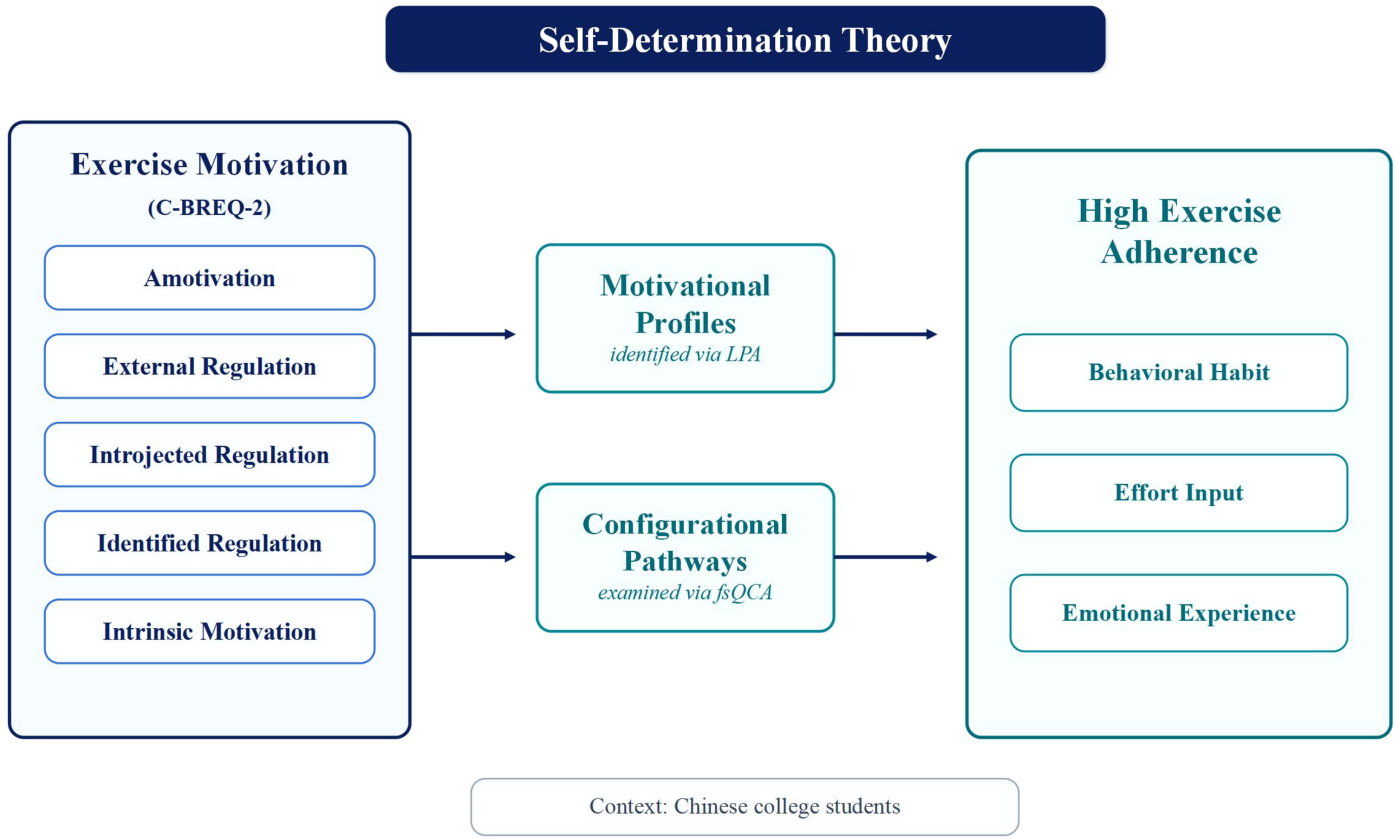
Theoretical model of motivational profiles and configurational pathways to high exercise adherence. The three EAQ dimensions shown under High Exercise Adherence—Behavioral Habit, Effort Input, and Emotional Experience—were averaged to create an overall exercise adherence score. In the subsequent analyses, this overall EAQ score, rather than the three separate EAQ dimensions, served as the adherence outcome.

Building on self-determination theory, the present study examines how different forms of exercise motivation are associated with high exercise adherence among Chinese college students (Figure 1). Specifically, it integrates LPA and fsQCA to provide a more comprehensive understanding of both motivational heterogeneity and configurational complexity. First, LPA is used to identify latent motivational profiles based on five types of exercise motivation: amotivation, external regulation, introjected regulation, identified regulation, and intrinsic motivation. Second, fsQCA is used to examine which configurations of these motivational conditions are sufficient for high exercise adherence.

This study is guided by the following research questions:

*RQ1*: What latent exercise motivation profiles can be identified among Chinese college students based on amotivation, external regulation, introjected regulation, identified regulation, and intrinsic motivation?

*RQ2*: How do the identified motivational profiles differ in terms of exercise adherence?

*RQ3*: Which configurations of exercise motivation conditions are sufficient for high exercise adherence among Chinese college students?

The study aims to make three contributions. First, theoretically, it extends the application of self-determination theory by moving beyond a purely variable-centered perspective and examining how different motivational regulations coexist within students. This helps reveal not only the quality of exercise motivation, but also its internal configuration. Second, methodologically, it combines LPA and fsQCA, allowing the study to identify both motivational profiles and set-theoretic configurations associated with high exercise adherence. This integrated design is particularly suitable for understanding complex health behaviors that may be produced by combinations of psychological conditions. Third, practically, the findings may provide more differentiated implications for exercise promotion in Chinese universities by showing which motivational profiles and configurations are more closely associated with sustained exercise adherence.

## 2 Materials and methods

### 2.1 Participants and procedure

This cross-sectional study was conducted among undergraduate and postgraduate students from 20 universities in Sichuan Province, China, including Sichuan University, Southwestern University of Finance and Economics, Southwest Jiaotong University, Southwest Petroleum University, Chengdu University, Sichuan Tourism University, Chengdu Sport University, and other higher education institutions. Data were collected between October 15, 2025 and December 15, 2025. Participants were recruited using Wenjuanxing, a widely used online survey platform in China, together with a snowball sampling approach. Specifically, the questionnaire link was initially distributed through university-related online groups, student communities, and peer networks. Participants were also encouraged to forward the survey link to eligible classmates or peers enrolled in universities in Sichuan Province.

The study protocol was reviewed and approved by the Ethics Committee of Sichuan Sports College, China (Approval No. SSC2025LL031). All procedures were conducted in accordance with the principles of voluntary participation, informed consent, anonymity, and confidentiality. Before accessing the formal questionnaire, participants were presented with an information sheet on the first page of the online survey. The information sheet explained the purpose of the study, the voluntary nature of participation, the approximate time required to complete the questionnaire, the confidentiality of responses, and participants’ right to withdraw from the study before submission without penalty. The first page also clearly stated that only individuals aged 18 years or older were eligible to participate. Participants were required to confirm that they were at least 18 years old and to provide electronic informed consent by clicking the consent confirmation option before proceeding to the questionnaire. Therefore, all participants included in the final sample were adults who had provided informed consent.

A total of 2,900 questionnaires were initially collected. Because all items in the online questionnaire were set as mandatory, there were no missing data in the raw dataset. To ensure data quality, responses were screened according to predefined exclusion criteria. Questionnaires were removed if they met any of the following conditions: completion time shorter than 180 s, which was considered insufficient for carefully reading and responding to all items; evident patterned or careless responding, such as selecting the same response option for nearly all Likert-type items or displaying highly repetitive response sequences; logically inconsistent responses to demographic or screening questions; or duplicate submissions identified through the survey platform’s duplicate-checking function. After data screening, 223 invalid questionnaires were excluded.

The final analytic sample consisted of 2,677 valid questionnaires, yielding an effective response rate of 92.31%. The sample included students from different grades, academic disciplines, and types of universities, thereby providing sufficient heterogeneity for examining exercise motivation profiles and their associations with high exercise adherence. The demographic characteristics of the final sample are presented in Table 1.

**TABLE 1:** Demographic characteristics of participants (*N* = 2,677).

Characteristic	Category	*n*	%
Gender	Male	1,320	49.31
	Female	1,357	50.69
Age	18–19 years	748	27.94
	20–21 years	1,096	40.94
	22–23 years	626	23.38
	≥ 24 years	207	7.73
Grade	First year	655	24.47
	Second year	724	27.05
	Third year	657	24.54
	Fourth year or above	641	23.94
University type	Comprehensive university	900	33.62
	Finance/economics university	336	12.55
	Engineering/science university	621	23.20
	Sport university/college	314	11.73
	Tourism/normal/other applied university	506	18.90
Major category	Humanities and social sciences	664	24.80
	Economics and management	523	19.54
	Science and engineering	736	27.49
	Medicine and health-related fields	209	7.81
	Physical education and sport-related majors	362	13.52
	Arts and other majors	183	6.84
Residence before university	Urban	1,485	55.47
	Rural	1,192	44.53
Monthly living expenses	< 1,000 RMB	326	12.18
	1,000–1,499 RMB	882	32.95
	1,500–1,999 RMB	831	31.04
	≥ 2,000 RMB	638	23.83
Exercise frequency	≤ 1 time/week	643	24.02
	2–3 times/week	1,094	40.87
	4–5 times/week	646	24.13
	≥ 6 times/week	294	10.98

### 2.2 Measurements

#### 2.2.1 Exercise motivation

Exercise motivation was measured using the Chinese-translated Behavioral Regulation in Exercise Questionnaire-2 (C-BREQ-2). The original Behavioral Regulation in Exercise Questionnaire-2 (BREQ-2) was developed within the framework of self-determination theory to assess different forms of behavioral regulation in exercise contexts. Markland and Tobin modified the original BREQ by reinstating amotivation items, thereby allowing the instrument to assess five forms of exercise motivation: amotivation, external regulation, introjected regulation, identified regulation, and intrinsic motivation (Markland and Tobin, 2004). The original BREQ-2 uses a 5-point response scale ranging from 0 = “not true for me” to 4 = “very true for me.”

Because the present study focuses on Chinese college students, the Chinese-translated version of the BREQ-2 was used. Liu et al. (2015) validated the C-BREQ-2 among university students from Mainland China and Hong Kong. Their results supported an 18-item, five-factor structure and provided evidence for factorial validity, discriminant validity, nomological validity, internal reliability, and measurement invariance across Mainland Chinese and Hong Kong university student samples. Therefore, the C-BREQ-2 is suitable for examining exercise motivation in Chinese university contexts (Liu et al., 2015).

The C-BREQ-2 contains five dimensions: Amotivation (4 items), External Regulation (4 items), Introjected Regulation (3 items), Identified Regulation (3 items), and Intrinsic Motivation (4 items). In the present study, each dimension score was calculated by averaging the items belonging to that dimension, with higher scores indicating a higher level of the corresponding type of exercise motivation. The five C-BREQ-2 subscale scores were therefore calculated as observed item mean scores. These observed subscale mean scores, rather than EFA- or CFA-derived factor scores, were used as indicators in the latent profile analysis and as condition variables in the subsequent fsQCA.

#### 2.2.2 Exercise adherence

Exercise adherence was measured using the Exercise Adherence Questionnaire (EAQ), developed by Wang et al. (2016). Exercise adherence refers to a persistent behavioral tendency demonstrated by individuals during physical exercise. The EAQ contains 14 items and consists of three dimensions: Behavioral Habit, Effort Input, and Emotional Experience. Participants responded to each item on a 5-point Likert scale ranging from 1 = “strongly disagree” to 5 = “strongly agree,” with higher scores indicating stronger exercise adherence.

The three dimensions capture different aspects of adherence to exercise. Behavioral Habit consists of 4 items and reflects the extent to which individuals have developed stable and regular exercise habits. Effort Input consists of 5 items and reflects the degree to which individuals invest time, energy, and persistence in maintaining exercise participation. Emotional Experience consists of 5 items and reflects the positive emotional experiences associated with exercise participation. The total exercise adherence score was calculated as the mean score of all 14 items. This overall score, rather than the three separate EAQ dimensions, was used as the distal outcome in the BCH analysis and as the fuzzy-set outcome of High Exercise Adherence in the fsQCA.

The EAQ has been widely used in Chinese educational and exercise psychology contexts. Previous studies using Chinese student samples have reported that the EAQ consists of 14 items across three dimensions—behavioral habit, effort input, and emotional experience—and have shown acceptable internal consistency and construct validity (Liu et al., 2024; Wang et al., 2016). These findings support the appropriateness of using the EAQ to assess exercise adherence among Chinese college students. Because the EAQ includes behavioral, effort-related, and emotional components, the total score should be interpreted as a self-reported adherence tendency rather than an objective measure of physical activity volume.

### 2.3 Data analysis

Data analyses were conducted using IBM SPSS Statistics 29.0, Mplus 8.3, and fsQCA 3.0. IBM SPSS Statistics 29.0 (IBM Corp, 2023) was used for data screening, descriptive statistics, reliability analysis, exploratory factor analysis for common method bias, and bivariate correlations. Mplus 8.3 (Muthén and Muthén, 1998–2017) was used for confirmatory factor analyses, latent profile analysis, and the Bolck, Croon, and Hagenaars (BCH) analysis of distal outcome differences. fsQCA 3.0 (Ragin and Davey, 2017) was used to calibrate fuzzy-set membership scores and to conduct necessary and sufficient condition analyses.

Before the main analyses, the dataset was screened according to the predefined data quality criteria described above. Because all questionnaire items were set as mandatory in the online survey system, there were no missing values. Descriptive statistics, including means and standard deviations, were calculated for all study variables. Internal consistency was assessed using Cronbach’s alpha, with values above 0.70 considered acceptable for research purposes (Cronbach, 1951; Nunnally and Bernstein, 1994). Composite reliability (CR) and average variance extracted (AVE) were calculated to evaluate construct reliability and convergent validity. CR values above 0.70 and AVE values above 0.50 were regarded as evidence of acceptable reliability and convergent validity (Fornell and Larcker, 1981; Hair et al., 2019). CR and AVE were calculated from the standardized factor loadings obtained from the corresponding CFA model for each scale. Specifically, for the C-BREQ-2, CR and AVE were calculated from the five-factor CFA; for the EAQ, CR and AVE were calculated from the three-factor CFA.

Because all variables were collected using self-reported questionnaires, common method bias was examined before conducting the main analyses. Harman’s single-factor exploratory factor analysis was conducted only as a diagnostic test for common method bias. It was not used to determine the factor structure of the C-BREQ-2 or the EAQ. Common method bias was considered unlikely to be a serious concern if multiple factors were extracted and the first unrotated factor did not account for the majority of the total variance (Podsakoff et al., 2003).

Separate confirmatory factor analyses were conducted for the C-BREQ-2 and the EAQ. The C-BREQ-2 was tested as an 18-item five-factor model, corresponding to amotivation, external regulation, introjected regulation, identified regulation, and intrinsic motivation. The EAQ was tested as a 14-item three-factor model, corresponding to behavioral habit, effort input, and emotional experience. A combined eight-factor model was additionally examined as a supplementary check of discriminant validity and common method bias, but the primary measurement evidence was based on the separate CFA models for each scale. Model fit was evaluated using multiple fit indices, including chi-square divided by degrees of freedom (χ^2^/df), comparative fit index (CFI), Tucker–Lewis index (TLI), standardized root mean square residual (SRMR), and root mean square error of approximation (RMSEA). In general, CFI and TLI values of 0.90 or above indicate acceptable fit, values of 0.95 or above indicate good fit, and SRMR and RMSEA values below 0.08 indicate acceptable fit (Hu and Bentler, 1999; Kline, 2016).

Convergent and discriminant validity were further examined after the measurement models were established. Standardized factor loadings were inspected to determine whether observed items adequately represented their corresponding latent constructs. Discriminant validity was first evaluated using the Fornell–Larcker criterion, according to which the square root of AVE for each construct should be greater than its correlations with other constructs (Fornell and Larcker, 1981). In addition, the heterotrait–monotrait ratio of correlations (HTMT) was calculated as a supplementary assessment of discriminant validity. HTMT values below 0.85 were considered evidence that the constructs were empirically distinct (Henseler et al., 2015). Pearson correlations were then used to examine bivariate associations among the five exercise motivation dimensions and the three exercise adherence dimensions.

Latent profile analysis was conducted in Mplus 8.3 to identify unobserved subgroups of students based on the five observed C-BREQ-2 subscale mean scores: amotivation, external regulation, introjected regulation, identified regulation, and intrinsic motivation. No EFA- or CFA-derived factor scores were used in the LPA. Models with different numbers of latent profiles were estimated and compared. The optimal profile solution was selected based on a combination of statistical fit indices, classification quality, profile size, parsimony, and theoretical interpretability. Specifically, the Akaike information criterion (AIC), Bayesian information criterion (BIC), and sample-size-adjusted Bayesian information criterion (aBIC) were used to compare model fit, with smaller values indicating better fit. Entropy was used to assess classification accuracy, with higher values indicating clearer classification. The Lo–Mendell–Rubin adjusted likelihood ratio test (LMR) and bootstrap likelihood ratio test (BLRT) were used to compare whether a k-profile model fit the data significantly better than a k - 1 profile model (Lo et al., 2001; Nylund et al., 2007). In addition to statistical indices, profile interpretability and the proportion of participants in each profile were considered to avoid over-extraction of small or theoretically unstable profiles.

After the optimal latent profile solution was determined, the Bolck, Croon, and Hagenaars (BCH) method was used to compare exercise adherence across latent profiles as a continuous distal outcome. The BCH approach was selected because it accounts for classification uncertainty and reduces bias when examining differences in distal outcomes across latent classes or profiles (Asparouhov and Muthén, 2014; Bakk and Vermunt, 2016). BCH-adjusted means and standard errors were estimated for each profile, and an overall Wald chi-square test was used to determine whether exercise adherence differed significantly across profiles. Pairwise comparisons were then conducted to identify specific differences between profiles. To control for multiple testing across pairwise comparisons, *p*-values were adjusted using the Holm-Bonferroni procedure (Holm, 1979).

Fuzzy-set qualitative comparative analysis was conducted using fsQCA 3.0 (Ragin and Davey, 2017) to examine which configurations of exercise motivation conditions were sufficient for high exercise adherence. The five observed C-BREQ-2 subscale mean scores were treated as causal conditions, and the overall EAQ score was treated as the outcome. Following the direct calibration approach, each condition and the outcome were transformed into fuzzy-set membership scores ranging from 0 to 1 using three qualitative anchors: full membership, crossover point, and full non-membership (Ragin, 2008, 2009). Calibration anchors were determined based on the scale properties, theoretical meaning of the constructs, and empirical distribution of the data. For exercise adherence, the calibrated set represented membership in the outcome condition of high exercise adherence.

The fsQCA proceeded in two stages. First, necessary condition analysis was conducted to determine whether any single motivational condition, or its absence, was necessary for high or non-high exercise adherence. Following established fsQCA guidelines, a condition was considered necessary only when its consistency exceeded 0.90, indicating that cases with the outcome were consistently members of the condition set (Ragin, 2008; Schneider and Wagemann, 2012). Second, sufficiency analysis was conducted through truth table analysis. The frequency threshold was set according to the sample size and the distribution of cases across configurations. The consistency threshold was determined based on recommended fsQCA standards and the empirical distribution of truth table consistency values. Proportional reduction in inconsistency (PRI) consistency was also considered to reduce the influence of contradictory configurations. The Quine–McCluskey algorithm was used to generate complex, parsimonious, and intermediate solutions. Following configurational analysis conventions, conditions appearing in both parsimonious and intermediate solutions were interpreted as core conditions, whereas conditions appearing only in intermediate solutions were interpreted as peripheral conditions (Fiss, 2011). To assess the stability of the configurational findings, robustness checks were conducted by adjusting key analytical parameters, including the frequency threshold and consistency threshold.

## 3 Results

### 3.1 Common method bias test

As all data were collected using self-reported questionnaires, common method bias was examined before conducting the main analyses. Harman’s single-factor exploratory factor analysis was conducted as a diagnostic test for common method bias. The results showed that eight factors with eigenvalues > 1 were extracted, accounting for 67.306% of the total variance. The first unrotated factor explained 30.625% of the total variance, which was lower than the commonly recommended threshold of 40%. This result suggested that common method bias was unlikely to seriously affect the findings of the present study.

As an additional supplementary check, a combined eight-factor model including the five exercise motivation dimensions and the three exercise adherence dimensions was compared with several alternative models in which theoretically adjacent constructs were progressively combined. These supplementary results are reported in Supplementary Table 1. The combined eight-factor model showed better fit than the one-factor model and other alternative combined models, further suggesting that the measured constructs were empirically distinguishable. However, this combined model was used only as a supplementary check of discriminant validity and common method bias; the primary measurement evidence for the two instruments was based on the separate CFA models reported below.

### 3.2 Measurement models

Separate confirmatory factor analyses were conducted to evaluate the measurement structures of the two instruments. The C-BREQ-2 was tested as an 18-item five-factor model, corresponding to amotivation, external regulation, introjected regulation, identified regulation, and intrinsic motivation. The EAQ was tested as a 14-item three-factor model, corresponding to behavioral habit, effort input, and emotional experience. As shown in Table 2, both the five-factor C-BREQ-2 model and the three-factor EAQ model showed acceptable-to-good model fit. These results supported the appropriateness of evaluating the two scales as theoretically distinct instruments.

**TABLE 2:** Confirmatory factor analysis results for the C-BREQ-2 and EAQ.

Scale	Model	χ ^2^	df	χ ^2^/df	CFI	TLI	SRMR	RMSEA [90% CI]
C-BREQ-2	Five-factor CFA	161.889	125	1.295	0.998	0.998	0.012	0.010 [0.005, 0.015]
EAQ	Three-factor CFA	321.092	74	4.339	0.986	0.983	0.021	0.035 [0.031, 0.039]

C-BREQ-2, Chinese-translated Behavioral Regulation in Exercise Questionnaire-2; EAQ, Exercise Adherence Questionnaire; CFI, comparative fit index; TLI, Tucker–Lewis index; SRMR, standardized root mean square residual; RMSEA, root mean square error of approximation. The C-BREQ-2 model specified five correlated factors: amotivation, external regulation, introjected regulation, identified regulation, and intrinsic motivation. The EAQ model specified three correlated factors: behavioral habit, effort input, and emotional experience.

### 3.3 Preliminary analyses

Preliminary analyses were conducted to examine descriptive statistics, reliability, convergent validity, discriminant validity, and bivariate correlations among the study variables. As shown in Table 3, the mean scores of the five exercise motivation dimensions ranged from 1.410 to 2.703, while the mean scores of the three exercise adherence dimensions ranged from 3.464 to 3.636. Specifically, amotivation and external regulation showed relatively low mean scores, whereas behavioral habit, effort input, and emotional experience showed relatively higher mean scores. The overall exercise adherence score, calculated as the mean of all 14 EAQ items, had a mean of 3.533 and a standard deviation of 0.764.

**TABLE 3:** Descriptive statistics, reliability, convergent validity, and correlations among the study variables.

Variable	*M*	SD	Cronbach’s α	Std. loading range	CR	AVE	AMOT	EXT	INTRO	IDEN	IM	BH	EI	EE
AMOT	1.433	0.997	0.863	0.759–0.845	0.864	0.615	**0.784**	**0.730**	**0.742**	**0.753**	**0.739**	**0.773**	**0.723**	**0.760**
EXT	1.410	0.898	0.820	0.709–0.744	0.820	0.533	0.469							
INTRO	2.571	0.877	0.785	0.727–0.766	0.786	0.550	–0.464	–0.441						
IDEN	2.703	0.919	0.783	0.726–0.780	0.797	0.568	–0.471	–0.434	0.447					
IM	2.664	0.951	0.825	0.679–0.782	0.828	0.546	–0.498	–0.500	0.445	0.453				
BH	3.636	0.931	0.854	0.736–0.825	0.855	0.597	–0.380	–0.327	0.263	0.169	0.367			
EI	3.519	0.920	0.841	0.698–0.774	0.845	0.522	–0.354	–0.340	0.267	0.169	0.306	0.482		
EE	3.464	0.979	0.871	0.742–0.816	0.872	0.577	–0.359	–0.324	0.247	0.160	0.319	0.474	0.478	

AMOT, amotivation; EXT, external regulation; INTRO, introjected regulation; IDEN, identified regulation; IM, intrinsic motivation; BH, behavioral habit; EI, effort input; EE, emotional experience; M, mean; SD, standard deviation; CR, composite reliability; AVE, average variance extracted. Std. loading range refers to the range of standardized factor loadings. Bold diagonal values represent the square roots of AVE, and off-diagonal values represent Pearson correlation coefficients. CR and AVE were calculated from the standardized factor loadings obtained from the corresponding CFA model for each scale. All bivariate correlations were statistically significant at *p* < 0.01.

The reliability of all constructs was satisfactory. Cronbach’s α values for the five exercise motivation dimensions and the three exercise adherence dimensions ranged from 0.783 to 0.871, exceeding the commonly recommended threshold of 0.70. The Cronbach’s α value for the overall 14-item EAQ score was 0.896, indicating good internal consistency for the overall exercise adherence measure. Based on the standardized factor loadings from the separate CFA models, composite reliability values ranged from 0.786 to 0.872, and average variance extracted values ranged from 0.523 to 0.615. These values met the recommended criteria for construct reliability and convergent validity. The standardized factor loadings ranged from 0.680 to 0.839, suggesting that the observed indicators adequately represented their corresponding latent constructs.

Discriminant validity was first assessed using the Fornell–Larcker criterion. As shown in Table 3, the square root of AVE for each construct was greater than the absolute value of its correlations with other constructs, indicating adequate discriminant validity among the eight constructs. The correlation results further showed that amotivation and external regulation were negatively associated with the three dimensions of exercise adherence, whereas introjected regulation, identified regulation, and intrinsic motivation were positively associated with behavioral habit, effort input, and emotional experience. All bivariate correlations were statistically significant at *p* < 0.01.

To further verify discriminant validity, the heterotrait–monotrait ratio of correlations was calculated. As shown in Table 4, all HTMT values ranged from 0.194 to 0.607, which were below the conservative threshold of 0.85. Therefore, the results provided additional evidence that the constructs measured in this study were empirically distinct. Taken together, the preliminary analyses indicated that the study variables had acceptable reliability, convergent validity, and discriminant validity, supporting their use in the subsequent latent profile analysis and fuzzy-set qualitative comparative analysis.

**TABLE 4:** Discriminant validity based on the heterotrait-monotrait ratio of correlations.

Variable	AMOT	EXT	INTRO	IDEN	IM	BH	EI	EE
AMOT	-	-	-	-	-	-	-	-
EXT	0.557							
INTRO	0.562	0.548						
IDEN	0.573	0.538	0.567					
IM	0.590	0.607	0.553	0.562				
BH	0.443	0.391	0.321	0.209	0.438			
EI	0.417	0.411	0.330	0.208	0.369	0.571		
EE	0.413	0.382	0.298	0.194	0.377	0.548	0.557	

AMOT, amotivation; EXT, external regulation; INTRO, introjected regulation; IDEN, identified regulation; IM, intrinsic motivation; BH, behavioral habit; EI, effort input; EE, emotional experience. Values below the diagonal represent the heterotrait-monotrait ratio of correlations. All HTMT values were below the conservative threshold of 0.85.

### 3.4 Latent profile analysis

Latent profile analysis was conducted using the five observed C-BREQ-2 subscale mean scores, namely amotivation, external regulation, introjected regulation, identified regulation, and intrinsic motivation. Models with one to five latent profiles were estimated and compared. The optimal number of profiles was determined based on a combination of statistical fit indices, classification quality, profile size, parsimony, and theoretical interpretability.

As shown in Table 5, the AIC, BIC, and aBIC values decreased continuously from the one-profile model to the five-profile model. The four-profile model showed good classification quality, with an entropy value of 0.813. In addition, both the LMR and BLRT values were significant for the four-profile model, indicating that the four-profile solution fit the data significantly better than the three-profile solution. Although the five-profile model showed slightly lower AIC, BIC, and aBIC values, its LMR test was not significant, *p* = 0.107, suggesting that the five-profile model did not provide a statistically significant improvement over the four-profile model. Moreover, the five-profile model contained a relatively small profile, with a profile probability of 0.057, which reduced its parsimony and theoretical interpretability. Therefore, the four-profile model was selected as the optimal solution.

**TABLE 5:** Model fit indices for one- to five-profile latent profile solutions.

No. of profiles	AIC	BIC	aBIC	Entropy	LMR (p)	BLRT (p)	Category probability	Log likelihood
1	35989.012	36047.936	36016.163	-	-	-	1.000	-17984.506
2	32027.345	32121.624	32070.787	0.837	<0.001	<0.001	0.558/0.442	-15997.672
3	31708.723	31838.537	31768.457	0.709	<0.001	<0.001	0.283/0.414/0.303	-15832.362
4	31307.889	31472.877	31383.913	0.813	<0.001	<0.001	0.167/0.253/0.425/0.155	-15625.944
5	31164.788	31365.131	31257.103	0.813	0.107	<0.001	0.254/0.130/0.057/0.139/0.420	-15548.394

AIC, Akaike information criterion; BIC, Bayesian information criterion; aBIC, sample-size-adjusted Bayesian information criterion; LMR, Lo–Mendell–Rubin adjusted likelihood ratio test; BLRT, bootstrap likelihood ratio test. Lower AIC, BIC, and aBIC values indicate better model fit, whereas higher entropy values indicate greater classification accuracy. Significant LMR and BLRT values indicate that the k-profile model fits significantly better than the k - 1 profile model. Profile proportions are based on the estimated latent profile probabilities. Entropy, LMR, and BLRT are not applicable to the one-profile model.

The characteristics and sample distribution of the four latent profiles are presented in Table 6. Profile 1 included 446 students, accounting for 16.66% of the sample. This profile was characterized by high intrinsic motivation, moderate introjected regulation, and relatively low amotivation and external regulation; therefore, it was labeled the intrinsic-dominant motivation profile. Profile 2 included 678 students, accounting for 25.33% of the sample. This profile showed relatively high amotivation and external regulation, together with low identified regulation and intrinsic motivation; therefore, it was labeled the amotivated–controlled motivation profile. Profile 3 was the largest group, including 1,138 students, accounting for 42.51% of the sample. This profile was characterized by very low amotivation and external regulation, but high introjected regulation, identified regulation, and intrinsic motivation; therefore, it was labeled the high autonomous motivation profile. Profile 4 included 415 students, accounting for 15.50% of the sample. This profile showed high identified regulation, moderate introjected and external regulation, and relatively low intrinsic motivation; therefore, it was labeled the identified-regulation dominant profile.

**TABLE 6:** Characteristics and sample distribution of latent exercise motivation profiles.

Profile	Profile label	*n*	%	AMOT M (SE)	EXT M (SE)	INTRO M (SE)	IDEN M (SE)	IM M (SE)
Profile 1	Intrinsic-dominant motivation profile	446	16.66	1.414 (0.092)	1.312 (0.072)	2.572 (0.075)	2.000 (0.043)	3.039 (0.055)
Profile 2	Amotivated–controlled motivation profile	678	25.33	2.353 (0.036)	2.189 (0.034)	1.842 (0.031)	1.658 (0.030)	1.695 (0.044)
Profile 3	High autonomous motivation profile	1,138	42.51	0.751 (0.023)	0.826 (0.022)	3.124 (0.024)	3.464 (0.019)	3.384 (0.029)
Profile 4	Identified-regulation dominant profile	415	15.50	1.799 (0.097)	1.822 (0.077)	2.262 (0.067)	3.090 (0.042)	1.899 (0.070)

AMOT, amotivation; EXT, external regulation; INTRO, introjected regulation; IDEN, identified regulation; IM, intrinsic motivation. Values are model-estimated class-specific means with standard errors in parentheses based on the original C-BREQ-2 subscale scores. All C-BREQ-2 subscales range from 0 to 4, with higher scores indicating higher levels of the corresponding motivational regulation. Class sizes (n and %) are based on most likely latent profile membership. Mplus class numbers were reordered to match the revised profile labels and the original profile pattern.

**FIGURE 2:**
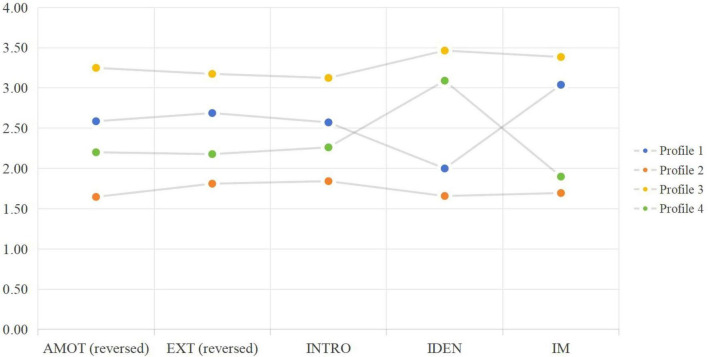
Estimated indicator means across the four latent exercise motivation profiles. AMOT, amotivation; EXT, external regulation; INTRO, introjected regulation; IDEN, identified regulation; IM, intrinsic motivation. Profile 1 = intrinsic-dominant motivation profile; Profile 2 = amotivated–controlled motivation profile; Profile 3 = high autonomous motivation profile; Profile 4 = identified-regulation dominant profile. For visualization purposes, AMOT and EXT were reverse-scored in this Figure, whereas INTRO, IDEN, and IM were plotted using their original values. The reversal of AMOT and EXT was conducted only for graphical presentation; all latent profile analyses, profile classification, and subsequent analyses were based on the original untransformed scores.

Figure 2 further illustrates the motivational patterns across the four profiles. To improve visual interpretability, amotivation and external regulation were reverse-scored only for the figure, so that higher plotted values consistently represented a more adaptive motivational pattern. This graphical transformation did not affect the latent profile analysis, profile classification, Table 6 values, or any subsequent analyses.

To examine whether exercise adherence differed across the four latent exercise motivation profiles, the Bolck, Croon, and Hagenaars (BCH) method was used with exercise adherence as a distal continuous outcome. As shown in Table 7, the overall Wald test indicated significant differences in exercise adherence across the four profiles, Wald χ^2^(3) = 637.468, *p* < 0.001. The BCH-adjusted means showed that Profile 3 had the highest numerical exercise adherence score, followed by Profile 1, whereas Profiles 2 and 4 showed relatively lower exercise adherence scores. After Holm-Bonferroni correction, Profile 1 had significantly higher exercise adherence than Profile 2 and Profile 4, and Profile 3 had significantly higher exercise adherence than Profile 2 and Profile 4. The difference between Profile 1 and Profile 3 was not significant after adjustment, and Profiles 2 and 4 also did not differ significantly from each other. Therefore, Profiles 1 and 3 showed relatively higher exercise adherence than Profiles 2 and 4, but the numerical difference between Profiles 1 and 3 should be interpreted cautiously.

**TABLE 7:** Differences in exercise adherence across latent exercise motivation profiles using the BCH method.

(A) BCH-adjusted exercise adherence means by profile.						
Profile	Profile label			EA M		SE
Profile 1	Intrinsic-dominant motivation profile			3.773		0.041
Profile 2	Amotivated–controlled motivation profile			3.093		0.029
Profile 3	High autonomous motivation profile			3.875		0.021
Profile 4	Identified-regulation dominant profile			3.072		0.044
**(B) Pairwise comparisons with Holm-Bonferroni adjustment.**						
**Comparison**	**Mean difference**	**SE**	**z**	**p_raw**	**p_Holm_ adjusted**	**Significance after Holm**
P1 vs. P2	0.680	0.053	12.828	<0.001	<0.001	Yes
P1 vs. P3	–0.103	0.048	–2.151	0.031	0.063	No
P1 vs. P4	0.701	0.062	11.216	<0.001	<0.001	Yes
P2 vs. P3	–0.783	0.036	–21.847	<0.001	<0.001	Yes
P2 vs. P4	0.020	0.055	0.363	0.717	0.717	No
P3 vs. P4	0.803	0.050	16.012	<0.001	<0.001	Yes

EA, exercise adherence. Values in panel A are BCH-adjusted means and standard errors. Overall Wald χ^2^(3) = 637.468, *p* < 0.001. P1 = Profile 1, intrinsic-dominant motivation profile; P2 = Profile 2, amotivated–controlled motivation profile; P3 = Profile 3, high autonomous motivation profile; P4 = Profile 4, identified-regulation dominant profile. Mean differences in panel B were calculated as the first profile minus the second profile in each comparison. Pairwise significance was determined using Holm-Bonferroni adjusted *p*-values.

### 3.5 Fuzzy-set qualitative comparative analysis

To further identify the combinations of motivational conditions associated with high and non-high exercise adherence, fuzzy-set qualitative comparative analysis was conducted. The five dimensions of exercise motivation, namely amotivation, external regulation, introjected regulation, identified regulation, and intrinsic motivation, were treated as causal conditions. Exercise adherence was treated as the outcome condition. Before conducting fsQCA, all conditions and the outcome were calibrated into fuzzy-set membership scores using the direct calibration method. For each variable, three qualitative anchors were specified, including full membership, crossover point, and full non-membership. The calibration anchors are shown in Table 8.

**TABLE 8:** Calibration anchors for fsQCA.

Set	Original variable	Full membership	Crossover point	Full non-membership
AMOT	Amotivation	3.250	1.250	0.000
EXT	External regulation	3.000	1.250	0.000
INTRO	Introjected regulation	4.000	2.667	1.000
IDEN	Identified regulation	4.000	3.000	1.000
IM	Intrinsic motivation	4.000	2.750	1.000
EA	Exercise adherence	4.650	3.633	2.250

AMOT, amotivation; EXT, external regulation; INTRO, introjected regulation; IDEN, identified regulation; IM, intrinsic motivation; EA, exercise adherence. The direct calibration method was used to transform raw scores into fuzzy-set membership scores. The three anchors represent full membership, crossover point, and full non-membership, respectively. For fsQCA, EA was calibrated as the fuzzy-set outcome of high exercise adherence.

A necessary condition analysis was first conducted to examine whether any single motivational condition, or its absence, was necessary for high or non-high exercise adherence. In fsQCA, a condition is interpreted as necessary when the outcome is consistently a subset of that condition. Following established QCA guidelines, consistency values above 0.90 were used as the criterion for necessity. As shown in Table 9, none of the five motivational conditions or their negations reached the recommended consistency threshold of 0.90 for either high exercise adherence or non-high exercise adherence. For high exercise adherence, the highest consistency values were observed for the absence of amotivation, intrinsic motivation, and the absence of external regulation, but their consistency values remained below 0.90. Similarly, for non-high exercise adherence, relatively high consistency values were observed for external regulation, amotivation, the absence of introjected regulation, and the absence of intrinsic motivation, but none reached the threshold for necessity. These findings indicate that no single motivational condition alone constituted a necessary condition for high or non-high exercise adherence. Therefore, it was appropriate to proceed with sufficiency analysis to identify how different motivational conditions combine to produce high or non-high exercise adherence.

**TABLE 9:** Necessary condition analysis for high and non-high exercise adherence (EA).

Condition	High EA		Non-high EA	
	Consistency	Coverage	Consistency	Coverage
AMOT	0.516	0.526	0.739	0.742
∼AMOT	0.747	0.744	0.527	0.518
EXT	0.548	0.544	0.743	0.728
∼EXT	0.726	0.741	0.535	0.539
INTRO	0.706	0.718	0.554	0.556
∼INTRO	0.563	0.562	0.719	0.707
IDEN	0.662	0.691	0.561	0.578
∼IDEN	0.595	0.579	0.699	0.671
IM	0.744	0.720	0.546	0.522
∼IM	0.506	0.531	0.707	0.732

“∼” denotes the absence or low level of a given condition. AMOT, amotivation; EXT, external regulation; INTRO, introjected regulation; IDEN, identified regulation; IM, intrinsic motivation; EA, exercise adherence. In fsQCA, a condition is generally considered necessary when its consistency exceeds 0.90.

After the necessary condition analysis, truth table analysis was conducted to identify sufficient configurations leading to high and non-high exercise adherence. Given the large sample size and the number of causal conditions, the frequency threshold was set to 10. The consistency threshold was determined based on both the recommended minimum standard and the distribution of consistency scores in the truth table. To reduce contradictory configurations, PRI consistency was also considered when coding truth table rows. The Quine–McCluskey algorithm was used to generate complex, parsimonious, and intermediate solutions. Because the complex, parsimonious, and intermediate solutions were identical in the present analysis, all conditions displayed in Table 10 were treated as core conditions.

**TABLE 10:** Configurational solutions for achieving high and non-high exercise adherence (EA).

Conditions	High EA S1	Non-high EA NS1
AMOT	⊗	●
EXT	⊗	●
INTRO		
IDEN		
IM	●	⊗
Consistency	0.841	0.858
Raw coverage	0.544	0.543
Unique coverage	0.544	0.543
Solution consistency	0.841	0.858
Solution coverage	0.544	0.543

● indicates the presence of a core condition; ⊗ indicates the absence of a core condition; blank cells indicate that the condition may be either present or absent. AMOT, amotivation; EXT, external regulation; INTRO, introjected regulation; IDEN, identified regulation; IM, intrinsic motivation; EA, exercise adherence. Because the complex, parsimonious, and intermediate solutions were identical, all displayed conditions were treated as core conditions.

As shown in Table 10, one sufficient configuration was identified for high exercise adherence. Although fsQCA allows for the possibility of multiple equifinal configurations, the present analysis identified one dominant sufficient configuration rather than multiple alternative sufficient configurations for high exercise adherence. This configuration consisted of the absence of amotivation, the absence of external regulation, and the presence of intrinsic motivation. The consistency and raw coverage of this configuration were 0.841 and 0.544, respectively. This result indicates that students characterized by low amotivation, low external regulation, and high intrinsic motivation were more likely to achieve high exercise adherence. In other words, a self-determined motivational pattern centered on intrinsic motivation, together with the absence of non-self-determined motivational states, was sufficient for high exercise adherence.

For non-high exercise adherence, one sufficient configuration was identified. This configuration consisted of the presence of amotivation, the presence of external regulation, and the absence of intrinsic motivation. The consistency and raw coverage of this configuration were 0.858 and 0.543, respectively. This finding suggests that students with high amotivation, high external regulation, and low intrinsic motivation were more likely to show non-high exercise adherence. The configurations for high and non-high exercise adherence formed a clear asymmetric pattern: high exercise adherence was explained by the combination of low amotivation, low external regulation, and high intrinsic motivation, whereas non-high exercise adherence was explained by the opposite combination of high amotivation, high external regulation, and low intrinsic motivation.

To examine the stability of the configurational results, robustness tests were conducted by systematically adjusting key analytical parameters. First, the frequency threshold was varied to examine whether the solutions were sensitive to the minimum number of cases required for retaining truth table rows. Second, the consistency threshold for identifying sufficient configurations was adjusted to test whether the results remained stable under stricter criteria. Third, PRI consistency was considered to reduce the influence of potentially contradictory configurations. Across these sensitivity checks, the core configurational structure remained unchanged.

Specifically, the configuration leading to high exercise adherence consistently involved the absence of amotivation, the absence of external regulation, and the presence of intrinsic motivation. Similarly, the configuration leading to non-high exercise adherence consistently involved the presence of amotivation, the presence of external regulation, and the absence of intrinsic motivation. These robustness checks indicate that the identified configurations were not sensitive to reasonable changes in analytical parameters. Therefore, the fsQCA results provide stable evidence that combinations of amotivation, external regulation, and intrinsic motivation play a central role in differentiating high from non-high exercise adherence.

## 4 Discussion

### 4.1 Overview of the main findings

Drawing on self-determination theory, the present study examined how different forms of exercise motivation are associated with exercise adherence among Chinese college students. By integrating latent profile analysis and fuzzy-set qualitative comparative analysis, this study provided evidence from both person-centered and configurational perspectives. Overall, the findings suggest that exercise adherence among college students is better understood by considering both motivational heterogeneity and motivational configuration.

First, the LPA identified four distinct exercise motivation profiles: the intrinsic-dominant motivation profile, the amotivated–controlled motivation profile, the high autonomous motivation profile, and the identified-regulation dominant profile. These profiles show that college students’ exercise motivation is not simply high or low, but consists of different combinations of amotivation, external regulation, introjected regulation, identified regulation, and intrinsic motivation. Second, the BCH analysis showed significant differences in exercise adherence across the four profiles. After Holm-Bonferroni correction, students in the high autonomous motivation profile and intrinsic-dominant motivation profile showed relatively higher exercise adherence than those in the amotivated–controlled motivation profile and identified-regulation dominant profile. However, the difference between the high autonomous motivation profile and intrinsic-dominant motivation profile was not significant after adjustment, and the amotivated–controlled motivation profile and identified-regulation dominant profile also did not differ significantly from each other. Third, the fsQCA results showed that no single motivational condition was necessary for high exercise adherence. Instead, high exercise adherence was associated with a set-theoretic configuration characterized by low amotivation, low external regulation, and high intrinsic motivation. Non-high exercise adherence was associated with the opposite pattern: high amotivation, high external regulation, and low intrinsic motivation.

Together, these findings suggest that exercise adherence among college students is closely related to both motivational profile and motivational configuration. The configurational analysis did not reveal multiple alternative routes; instead, it identified a concise self-determined configuration centered on low amotivation, low external regulation, and high intrinsic motivation. This pattern indicates that sustained exercise adherence may depend not only on strengthening adaptive motivation, but also on reducing non-self-determined motivational states.

### 4.2 Motivational heterogeneity among college students: evidence from latent profiles

The identification of four latent motivation profiles indicates clear motivational heterogeneity among Chinese college students. This finding is broadly consistent with previous person-centered research showing that exercise motivation is multidimensional and that autonomous, controlled, and amotivated regulations can combine into distinct motivational profiles. Lindwall et al. (2017), for example, identified within-person latent profiles of exercise motivation and showed that different behavioral regulations for exercise can coexist in heterogeneous motivational patterns. Rodrigues et al. (2019) also emphasized the importance of less self-determined or “dark-side” motivational processes for understanding intention to continue exercise. The present study extends this line of work by focusing on Chinese college students and by linking motivational profiles not only to exercise adherence, but also to configurational patterns associated with high adherence.

The high autonomous motivation profile represented one of the most adaptive patterns in this study. Students in this profile showed very low amotivation and external regulation, together with high introjected regulation, identified regulation, and intrinsic motivation. This pattern suggests that many students in this group experienced exercise as both personally meaningful and enjoyable, while being less dependent on external pressure. The intrinsic-dominant motivation profile also showed a relatively adaptive structure, especially because of its high intrinsic motivation and low non-self-determined motivation. However, compared with the high autonomous motivation profile, its identified regulation was lower, suggesting that enjoyment was more prominent than broader value internalization.

The amotivated–controlled motivation profile represented a less adaptive pattern, with relatively high amotivation and external regulation and low autonomous motivation. Students in this profile may experience exercise as weakly meaningful or externally required. The identified-regulation dominant profile is especially noteworthy. These students showed relatively high identified regulation, but lower intrinsic motivation and moderate controlled motivation. This means that they may recognize the value of exercise, but do not necessarily experience exercise itself as enjoyable or self-rewarding. This profile reflects a central issue in the present study: knowing that exercise is important does not necessarily correspond to strong exercise adherence.

Thus, the LPA findings extend self-determination theory by showing that the quality of exercise motivation should be understood not only at the level of separate motivational regulations, but also at the level of motivational profiles. The results also suggest that motivational profiles are not merely quantitative differences in overall motivation level; rather, they reflect qualitatively different patterns in how motivational regulations are combined within students.

### 4.3 Motivation quality and exercise adherence

The BCH results showed that exercise adherence differed significantly across the four motivational profiles. After Holm-Bonferroni correction, students in the high autonomous motivation profile and intrinsic-dominant motivation profile showed significantly higher exercise adherence than students in the amotivated–controlled motivation profile and identified-regulation dominant profile. However, the difference between the high autonomous motivation profile and intrinsic-dominant motivation profile was not significant after correction, and the amotivated–controlled motivation profile and identified-regulation dominant profile also did not differ significantly from each other. Therefore, the results should be interpreted as indicating two relatively higher-adherence profiles and two relatively lower-adherence profiles, rather than a fully ordered hierarchy among all four profiles.

This pattern suggests that exercise adherence is more closely associated with autonomous and intrinsically meaningful motivational structures than with value recognition alone (Mullan and Markland, 1997; Teixeira et al., 2012). Students in the high autonomous motivation profile were characterized by high intrinsic motivation and identified regulation, together with low amotivation and external regulation. Such a pattern combines enjoyment, personal value, and relatively low external control. Students in the intrinsic-dominant motivation profile also showed high intrinsic motivation and low non-self-determined motivation, which may explain why their adherence level was comparable to that of students in the high autonomous motivation profile after multiple-comparison adjustment.

The amotivated–controlled profile requires a cautious interpretation. Although this profile showed a less adaptive motivational pattern, its adherence score was not extremely low. This suggests that some students may continue exercising despite weak autonomous motivation, possibly because of course requirements, peer contexts, institutional expectations, or established routines. Therefore, this profile may represent externally maintained or structurally supported adherence rather than simple non-participation. This interpretation is consistent with the idea that controlled or non-self-determined motivation may sometimes maintain short-term participation, even if it is less favorable for high-quality and sustained engagement.

The identified-regulation dominant profile also deserves particular attention. Identified regulation is generally regarded as a relatively autonomous form of motivation, but in this study, high identified regulation was not accompanied by high intrinsic motivation. This suggests that recognizing the value or necessity of exercise may be different from enjoying exercise itself. These students may cognitively endorse exercise as important, but may not experience it as enjoyable, interesting, or self-rewarding. For college students, value recognition may support the intention to exercise, whereas intrinsic motivation may be more closely associated with sustained engagement and positive exercise experience (Marques et al., 2025; Teixeira et al., 2012). Therefore, the distinction between “exercise is useful” and “exercise is enjoyable” is theoretically important for understanding exercise adherence.

At the same time, the interpretation of intrinsic motivation should remain cautious because the EAQ includes an emotional experience component. The strong role of intrinsic motivation may therefore reflect both motivational quality and the affective experience of exercise adherence. Rather than treating this as a weakness alone, this finding also suggests that affective experience may be an important bridge between motivation and persistence. Future research should further distinguish behavioral persistence from emotional exercise experience and examine whether intrinsic motivation predicts objectively measured exercise behavior beyond self-reported adherence.

### 4.4 Configurational patterns associated with high exercise adherence

The fsQCA results further clarified the configurational pattern associated with high exercise adherence. The necessary condition analysis showed that no single motivational condition, nor its absence, reached the recommended threshold for necessity. This finding indicates that high exercise adherence was not linked to any single motivational condition alone. Rather, the configurational analysis showed that high exercise adherence was associated with the joint pattern of low amotivation, low external regulation, and high intrinsic motivation.

Although fsQCA allows for the possibility of equifinality, the present analysis identified one dominant sufficient configuration rather than multiple alternative pathways. This finding suggests that, in this sample, high exercise adherence was most consistently characterized by a self-determined motivational pattern: low amotivation, low external regulation, and high intrinsic motivation. In other words, the most stable configuration for high adherence was not simply the presence of autonomous motivation, but the simultaneous presence of intrinsic motivation and the relative absence of amotivation and external regulation.

This configuration is meaningful because it highlights both the presence of a positive motivational element and the relative absence of non-self-determined motivational states (Thøgersen-Ntoumani and Ntoumanis, 2006). Low amotivation suggests that students did not experience exercise as meaningless or irrelevant. Low external regulation suggests that their exercise behavior was not mainly characterized by external pressure or external requirements. High intrinsic motivation indicates that exercise was experienced as interesting, enjoyable, or personally satisfying (Luria, 2022). In combination, these conditions correspond to a more self-determined motivational pattern.

The configuration associated with non-high exercise adherence showed the opposite structure: high amotivation, high external regulation, and low intrinsic motivation. This pattern suggests that students with weaker enjoyment, weaker personal meaning, and stronger external regulation were more likely to belong to the non-high adherence set. Importantly, introjected regulation and identified regulation did not appear in the final configurations for either high or non-high exercise adherence. This does not mean that they are unimportant. Rather, it suggests that in the present set-theoretic analysis, amotivation, external regulation, and intrinsic motivation were more central in distinguishing high from non-high exercise adherence.

### 4.5 Integrating LPA and fsQCA: from student types to motivational configurations

The integration of LPA and fsQCA is one of the main strengths of this study. LPA identified different types of students based on their motivational profiles, whereas fsQCA identified motivational configurations associated with membership in high or non-high exercise adherence. These two methods therefore provide complementary perspectives.

The LPA results showed that students differ in how multiple motivational regulations are combined within individuals. The fsQCA results further showed that high exercise adherence was associated with a concise configuration involving low amotivation, low external regulation, and high intrinsic motivation. These two sets of findings are consistent with each other. The high autonomous motivation profile and intrinsic-dominant motivation profile both showed relatively low amotivation and external regulation and relatively high intrinsic motivation, and these profiles also showed higher exercise adherence than the amotivated–controlled motivation profile and identified-regulation dominant profile.

This combined approach avoids reducing exercise motivation to isolated variables or simple high-low distinctions. It suggests that exercise adherence among college students is better understood by considering both who students are motivationally and how different motivational conditions are configured. In this sense, the present study provides a more precise account of how motivational quality, profile membership, and configurational patterns are jointly associated with sustained exercise adherence.

### 4.6 Theoretical implications

This study has several theoretical implications. First, it extends the application of self-determination theory by showing that motivational quality can be examined through both profiles and configurations. The theoretical contribution of this study is not merely the identification of motivation profiles, as previous exercise motivation studies have already demonstrated motivational heterogeneity (Lindwall et al., 2017). Rather, the contribution lies in linking these profiles with exercise adherence among Chinese college students and further examining whether specific motivational configurations are sufficient for high adherence. This approach helps clarify how different forms of motivation coexist within students and how these combinations are associated with sustained exercise behavior.

Second, the findings refine the distinction between identified regulation and intrinsic motivation. The identified-regulation dominant profile showed that recognizing the value of exercise was not necessarily accompanied by high adherence when intrinsic motivation was relatively low and controlled motivation remained present. This suggests that identified regulation and intrinsic motivation may play different roles in exercise adherence. Identified regulation reflects perceived value, whereas intrinsic motivation reflects enjoyment and interest. The identified-regulation dominant profile therefore shows that value recognition alone may be insufficient when exercise is not experienced as enjoyable. This refines the SDT-based interpretation of adherence by distinguishing cognitive endorsement from affective enjoyment.

Third, the study highlights the importance of considering low amotivation and low external regulation, not only high autonomous motivation. The fsQCA results showed that high exercise adherence was associated with high intrinsic motivation together with low amotivation and low external regulation. This suggests that the absence of non-self-determined motivational states is an important part of the motivational pattern related to exercise adherence. Theoretically, this finding supports a more balanced interpretation of exercise motivation: adherence is associated not only with what motivates students, but also with what motivational barriers are relatively weak.

Fourth, the findings suggest that the affective and experiential aspects of exercise deserve more theoretical attention. Because the EAQ includes an emotional experience dimension, the association between intrinsic motivation and exercise adherence may partly reflect the affective quality of students’ exercise experiences. This does not weaken the relevance of intrinsic motivation; rather, it indicates that enjoyment, interest, and positive exercise experience may be central mechanisms through which motivation becomes sustained adherence. This point is consistent with affect-based perspectives on physical activity, which emphasize that how people feel during exercise can shape their willingness to continue future activity (Rhodes and Kates, 2015; Teixeira et al., 2024).

Finally, the study contributes methodologically by combining person-centered and configurational approaches. LPA revealed motivational heterogeneity, whereas fsQCA described set-theoretic motivational patterns associated with high and non-high exercise adherence. This combination offers a useful way to study complex health behaviors in which multiple psychological conditions coexist within individuals.

### 4.7 Practical implications for promoting exercise adherence in universities

The findings suggest that university exercise promotion should not rely on a single uniform strategy (Gao et al., 2025). Because students showed different motivational profiles, interventions may be more appropriate when they are matched to students’ motivational characteristics. At the same time, the fsQCA results suggest a common motivational target across profiles: reducing amotivation and external control while strengthening intrinsic motivation.

These practical implications are consistent with affect-based approaches to physical activity promotion, which emphasize that positive affect, enjoyment, and pleasant exercise experiences are important for sustaining physical activity. Rhodes and Kates (2015) showed that affective responses to exercise are relevant to future physical activity behavior and motivational processes. More recently, Teixeira et al. (2024) showed that targeting pleasant affective responses during exercise can improve short-term exercise attendance. Therefore, university exercise programs should not rely only on health education, compulsory participation, or fitness assessment, but should also create exercise experiences that students perceive as enjoyable, self-chosen, and emotionally rewarding.

For students in the high autonomous motivation profile, universities may focus on maintenance and enrichment. These students already showed an adaptive motivational pattern and relatively high adherence (Liang and Wang, 2026). Flexible exercise opportunities, diverse sport options, student-led clubs, open-access facilities, and self-improvement-oriented activities may be suitable for maintaining their autonomy and enjoyment. For this group, the goal is not simply to increase participation, but to preserve the motivational quality that already supports adherence.

For students in the intrinsic-dominant motivation profile, the key issue is to connect enjoyment with more stable personal meaning. These students may already enjoy exercise, but their identified regulation was not as strong as that of students in the high autonomous motivation profile. Therefore, physical education courses and campus health programs may include goal-setting, self-monitoring, and short reflective activities that encourage students to connect exercise with stress regulation, sleep quality, health management, and long-term wellbeing (Li et al., 2026). Such strategies may help transform enjoyable participation into more stable and personally meaningful adherence.

For students in the amotivated–controlled motivation profile, the goal should not be simply to increase exercise requirements. Strategies based mainly on pressure, compulsory attendance, or punitive assessment may reinforce the sense that exercise is externally imposed. Instead, interventions should reduce the sense of external compulsion and help students experience small, achievable, and positive exercise episodes. This group may need low-threshold and beginner-friendly opportunities, such as non-competitive activities, small-group exercise, peer companionship, and gradual skill-building experiences. The priority is to make exercise feel less externally imposed and more accessible, meaningful, and manageable.

For students in the identified-regulation dominant profile, additional health-benefit messages may be redundant because these students already recognize that exercise is important. The key intervention target is to transform value recognition into enjoyable experience by increasing choice, variety, peer support, and positive affect during exercise. Universities may provide more interest-based options, such as badminton, dance, yoga, cycling, fitness training, frisbee, recreational games, or other activities that allow students to find personally enjoyable forms of movement. Peer-based sport groups and supportive campus exercise communities may also be relevant for increasing the social and emotional appeal of exercise.

Overall, university exercise promotion should combine structure with autonomy support. Institutional arrangements such as physical education requirements, exercise check-ins, or fitness assessments may provide organization, but they should be implemented in ways that preserve choice, provide informational feedback, and emphasize personal progress rather than external control alone (Chu et al., 2019). A practical implication of the present findings is that universities should not only tell students why exercise is valuable, but also help them experience exercise as enjoyable, self-endorsed, and personally meaningful.

### 4.8 Limitations and future research

Several limitations should be noted. First, this study used a cross-sectional design. Although meaningful associations were found among motivational profiles, motivational configurations, and exercise adherence, causal conclusions cannot be drawn. Future research could use longitudinal designs to examine how motivational profiles change over time and how such changes are associated with later exercise adherence.

Second, all variables were measured using self-reported questionnaires. Although common method bias tests indicated that common method bias was unlikely to seriously affect the findings, self-report data may still be influenced by memory bias, social desirability, or subjective perceptions of exercise behavior. Future studies could combine questionnaire data with more objective or semi-objective indicators, such as accelerometer data, IPAQ-based activity estimates, exercise app records, wearable device data, campus gym entry records, physical education attendance records, or repeated weekly exercise logs.

Third, the EAQ includes an emotional experience dimension. Therefore, the total adherence score may partly reflect students’ affective experiences during exercise, not only their behavioral persistence. This issue is especially relevant because intrinsic motivation and emotional experience are conceptually related. Future studies should distinguish behavioral adherence from affective exercise experience more explicitly and examine whether the same motivational profiles and configurations predict objective or semi-objective indicators of exercise persistence.

Fourth, the inclusion of students from sport universities or sport-related majors should be considered when interpreting the findings. For these students, exercise may be part of daily training, academic requirements, or professional preparation rather than a purely voluntary leisure behavior. This may partly elevate adherence scores or strengthen identified and external regulation. It may also help explain why some students with less adaptive motivational profiles still reported moderate levels of adherence. Future studies should compare sport-related and non-sport-related students separately to examine whether the motivational profiles and configurational patterns are consistent across these groups.

Fifth, the sample was drawn from 20 universities in Sichuan Province. Although the sample was large and included students from different universities, grades, majors, and university types, the findings may not fully represent college students from other regions. Future research could examine whether the four-profile structure and configurational patterns are similar across different provinces, institutional types, and campus sport environments.

Sixth, the fsQCA model included only the five motivational dimensions of the C-BREQ-2. This was consistent with the theoretical focus of the study, but exercise adherence may also be associated with contextual factors such as facility accessibility, peer support, teacher autonomy support, academic workload, prior sport experience, perceived competence, and campus sport culture. Future studies could include both motivational and contextual conditions to examine more comprehensive configurational patterns.

Despite these limitations, the present study provides useful evidence for understanding exercise adherence among Chinese college students. The findings suggest that exercise adherence is closely associated with both motivational profiles and motivational configurations. In particular, high exercise adherence was more evident among students characterized by low amotivation, low external regulation, and high intrinsic motivation. This highlights the importance of creating university exercise environments that are not only structured, but also autonomy-supportive, enjoyable, and personally meaningful.

## 5 Conclusion

This study examined how different forms of exercise motivation are associated with high exercise adherence among Chinese college students by integrating latent profile analysis and fuzzy-set qualitative comparative analysis. The findings showed that students’ exercise motivation was heterogeneous and could be classified into four distinct profiles: intrinsic-dominant motivation profile, amotivated–controlled motivation profile, high autonomous motivation profile, and identified-regulation dominant profile. Students with high autonomous or intrinsic-dominant motivational patterns demonstrated relatively higher exercise adherence than those with amotivated–controlled or identified-regulation dominant patterns, indicating that sustained exercise behavior is more likely when intrinsic motivation is salient and non-self-determined motivation is relatively low. The fsQCA results further showed that high exercise adherence was not produced by any single motivational condition alone, but by a specific configuration involving the absence of amotivation, the absence of external regulation, and the presence of intrinsic motivation. Conversely, non-high exercise adherence was associated with the presence of amotivation, the presence of external regulation, and the absence of intrinsic motivation. These findings extend self-determination theory by demonstrating that exercise adherence is shaped by both motivational profiles and motivational configurations. Practically, the results suggest that universities should move beyond uniform exercise promotion strategies and create exercise environments that reduce external control and amotivation while strengthening enjoyment, autonomy, and personally meaningful engagement.

## Data Availability

The original contributions presented in this study are included in the article/Supplementary material, further inquiries can be directed to the corresponding author.
