# Supplementary material for: From “wanting to exercise” to “sticking with exercise”: motivational profiles and configurational pathways to high exercise adherence among college students—an LPA and fsQCA analysis
Source: Front Psychol. 2026 Jul 27;17:1886854. doi: 10.3389/fpsyg.2026.1886854 (PMC13455590; doi:10.3389/fpsyg.2026.1886854)
Supplement: Supplementary file 1 [file Table_1.docx]

Supplementary Table S1

*Supplementary comparison of combined measurement models for discriminant validity and common method bias*

| **Model** | **Factor structure** | **χ²** | **df** | **χ²/df** | **CFI** | **TLI** | **SRMR** | **RMSEA [90% CI]** |
| --- | --- | --- | --- | --- | --- | --- | --- | --- |
| Eight-factor model | AMOT, EXT, INTRO, IDEN, IM, BH, EI, EE | 709.166 | 436 | 1.627 | 0.993 | 0.992 | 0.017 | 0.015 [0.013, 0.017] |
| Seven-factor model | AMOT, EXT, INTRO, IDEN + IM, BH, EI, EE | 2312.607 | 443 | 5.220 | 0.952 | 0.947 | 0.034 | 0.040 [0.038, 0.041] |
| Six-factor model | AMOT, EXT + INTRO, IDEN + IM, BH, EI, EE | 3738.410 | 449 | 8.326 | 0.916 | 0.907 | 0.042 | 0.052 [0.051, 0.054] |
| Five-factor model | AMOT, EXT + INTRO, IDEN + IM, BH + EI, EE | 6166.078 | 454 | 13.582 | 0.854 | 0.841 | 0.052 | 0.069 [0.067, 0.070] |
| Four-factor model | AMOT, EXT + INTRO, IDEN + IM, BH + EI + EE | 8791.032 | 458 | 19.194 | 0.787 | 0.770 | 0.063 | 0.082 [0.081, 0.084] |
| Three-factor model | AMOT, EXT + INTRO + IDEN + IM, BH + EI + EE | 9810.169 | 461 | 21.280 | 0.762 | 0.743 | 0.066 | 0.087 [0.086, 0.089] |
| Two-factor model | AMOT + EXT + INTRO + IDEN + IM, BH + EI + EE | 11764.297 | 463 | 25.409 | 0.712 | 0.691 | 0.072 | 0.096 [0.094, 0.097] |
| One-factor model | AMOT + EXT + INTRO + IDEN + IM + BH + EI + EE | 17882.957 | 464 | 38.541 | 0.556 | 0.525 | 0.107 | 0.118 [0.117, 0.120] |

Note. AMOT = amotivation; EXT = external regulation; INTRO = introjected regulation; IDEN = identified regulation; IM = intrinsic motivation; BH = behavioral habit; EI = effort input; EE = emotional experience. The eight-factor model specified the five dimensions of exercise motivation and the three dimensions of exercise adherence as distinct but correlated latent factors. The alternative models were specified by progressively combining theoretically adjacent constructs. This combined model comparison was used only as a supplementary check of discriminant validity and common method bias; the primary measurement evidence was based on the separate CFA models for the C-BREQ-2 and EAQ.
